# Impact of enzyme replacement therapy on clinical manifestations in females with Fabry disease

**DOI:** 10.1186/s13023-024-03503-4

**Published:** 2024-12-27

**Authors:** Malte Lenders, Albina Nowak, Markus Cybulla, Jessica Kaufeld, Anja Friederike Köhn, Nicole Maria Muschol, Christine Kurschat, Eva Brand

**Affiliations:** 1 Department of Internal Medicine D, and Interdisciplinary Fabry Center (IFAZ), University Hospital Muenster, Muenster, Germany; 2 Department of Endocrinology and Clinical Nutrition, University Hospital Zuerich and University of Zuerich, Zuerich, Switzerland; 3 Department of Nephrology and Rheumatology, FGM, Center of Internal Medicine, Müllheim, Germany; 4 Department of Nephrology and Hypertension, Hannover Medical School, Hannover, Germany; 5 International Center for Lysosomal Disorders (ICLD), Department of Pediatrics, University Medical Center Hamburg-Eppendorf, Hamburg, Germany; 6 Department II of Internal Medicine, Center for Molecular Medicine Cologne and Center for Rare Diseases, University of Cologne, Cologne, Germany

**Keywords:** Females, Fabry disease, Treatment, Guidelines, lyso-Gb_3_

## Abstract

**Background:**

The aim of our multicenter study was to investigate the implementation of the European Fabry guidelines on therapeutic recommendations in female patients with Fabry disease (FD) and to analyze the impact of enzyme replacement therapy (ERT) in treated and untreated females.

**Results:**

Data from 3 consecutive visits of 159 female FD patients from 6 Fabry centers were retrospectively analyzed. According to their treatment, patients were separated in 3 groups (untreated, *n* = 71; newly ERT-treated, *n* = 47; long-term ERT-treated, *n* = 41). Clinical presentation and laboratory data, including plasma globotriaosylsphingosine (lyso-Gb_3_) levels were assessed. The observation time ranged from 49 to 62 months. ∼90% of female patients treated with ERT presented with at least one organ manifestation justifying treatment according to current European guidelines. Untreated females showed a less severe disease load with less FD-typical organ damage. All groups presented with a stable cardiac status (all *p* > 0.05) over time. ERT-treated females presented with a slight yearly loss of estimated glomerular filtration (eGFR) over time (both *p* < 0.05), which was comparable to the natural decline for this age. Plasma lyso-Gb_3_ levels were higher in ERT-treated females and decreased by 0.95 [-4.44 to 4.08] ng/ml/year (*p* = 0.0002) in those who were newly ERT-treated.

**Conclusions:**

Severely affected females with FD who were treated with ERT, and less severely affected untreated females, showed a broadly stable disease course over 5 years. The treatment decisions were largely based on the European guidelines for FD. In untreated females, it is crucial to explore if organ involvement is FD-related in order to make the correct treatment decision.

**Supplementary Information:**

The online version contains supplementary material available at 10.1186/s13023-024-03503-4.

## Introduction

Fabry disease (FD; OMIM #301500) is an X-chromosomal-linked lysosomal storage disease resulting from a deficient α-galactosidase A (AGAL) activity due to pathogenic variants within the respective *GLA* gene. FD-specific manifestations originate from systemic cellular lysosomal accumulation of mainly globotriaoslyceramide (Gb_3_) [1]. The progressive lysosomal accumulation results in a high risk of an early onset of stroke, life-threatening arrhythmia, myocardial infarction, or cardiac and renal failure, leading to a reduced life expectancy in males and females [1].

In general, females are heterozygous for the pathogenic *GLA* mutation and show X-chromosomal inactivation [2]. Therefore, they exhibit broader clinical phenotypic variability compared to males [2]. Female FD patients can be asymptomatic or exhibit often mild symptoms and typically present with symptoms and manifestations later than males. Current FD guidelines and recommendations in Europe suggest FD-specific treatment initiation in females with FD after the onset of first FD-typical renal, cardiac, and/or cerebral manifestations, or if FD-related pain or severe gastrointestinal complaints are present [3–5]. According to these guidelines, enzyme replacement therapy (ERT) should be considered in females with classical and non-classical phenotypes exhibiting albuminuria/proteinuria, an estimated glomerular filtration rate (eGFR) < 90 ml/min/1.73 m^2^, cardiac hypertrophy, signs of cardiac rhythm disturbances, cerebral white matter lesions, transient ischemic attack (TIA) or stroke, or Fabry-typical neuropathic pain or severe gastrointestinal complaints that are refractory to symptomatic therapy [4, 5]. Since ERT is assumed to be most effective when started early before the onset of fibrosis or other irreversible tissue damage [6–9], this strategy might result in a therapeutic dilemma.

In a previous study [10], we aimed to assess manifestations of and applied treatment concepts for females with FD according to the current European Fabry Guidelines. To this end, between 10/2008 and 12/2014, data from the most recent visit of 261 adult female FD patients from six German Fabry centers were retrospectively analyzed. In this study, we concluded that the treatment concept for females with FD in Germany was in line with the current European Fabry guidelines. However, a relevant number of females (one third) were untreated despite organ involvement, necessitating a careful reevaluation of these females [10]. Furthermore, since manifestations and symptoms in females are more heterogeneous compared with classic males, more longitudinal data for females are required to analyze the effect of ERT in these patients [2] or to exclude a potential disease progression in untreated females.

In the current study, we addressed our previous shortcomings and evaluated a cohort of 159 genetically confirmed female FD patients from six Fabry centers (five German centers, one Swiss center) with well-characterized clinical phenotypes at three time points to assess the implementation of current FD guidelines for treatment initiation with agalsidase-alfa or agalsidase-beta (Supplemental Table 1), the clinical impact of ERT in affected females and the disease course in untreated females over time.

## Methods

**Fig. 1:**
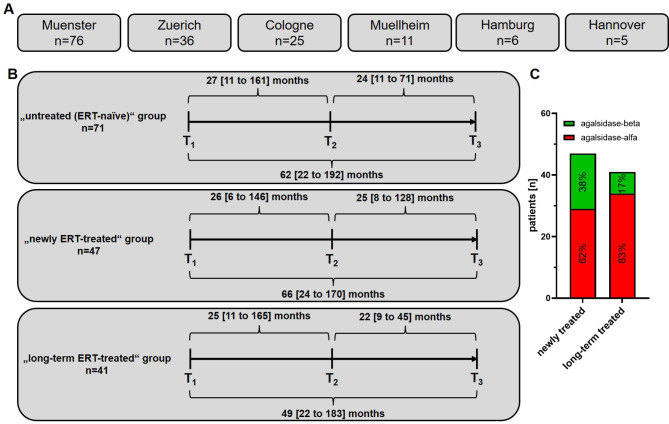
Overview of the study design. (**A**) In total, 159 female patients with genetically confirmed Fabry disease (FD) were consecutively recruited in five FD centers from Germany and one FD center from Switzerland. (**B**) Overview of the 3 analyzed groups and the median follow-up durations between 3 consecutive visits and overall observation periods with ranges. (**C**) Distribution of agalsidase-alfa and agalsidase-beta in both groups treated with enzyme replacement therapy

A total of 159 genetically confirmed adult female FD patients were consecutively recruited at the German Fabry centers of the University Hospitals in Muenster, Cologne, Hamburg and Hannover, as well as in the Fabry Center in Muellheim and Zuerich in Switzerland (Fig. 1A). All investigations were performed after approval by the respective ethics committees of the participating centers (project number: 2016-401-f-S; 2011-347-f) and written informed consent for molecular analysis and publication was obtained from all patients, where appropriate.

Patients were retrospectively analyzed at three time points (T1, T2, T3) (Fig. 1B). Inclusion criteria for this study were as follows: (i) female patient ≥ 18 years at time point T3 and a genetically confirmed disease-causing *GLA* mutation, (ii) naïve to any ERT or on a stable dose of agalsidase-alfa (0.2 mg/kg body weight) or agalsidase-beta (1.0 mg/kg body weight) for at least 6 months at time point T1, (iii) an interval of at least 6 months between visits (T1 to T2 and T2 to T3), (iv) patient currently not in any clinical trial. Since this study focused on the treatment effects of ERT (agalsidase-alfa and -beta) in females, patients receiving migalastat were not included. Furthermore, patients with mutations of unknown significance including p.S126G, p.A143T, and p.D313Y were also not included. A detailed overview of all detected *GLA* mutations is provided within Supplemental Table 2. Nonsense mutations were defined as single nucleotide exchanges, resulting in stop codons (termination), deletions or insertions of nucleotides resulting in a frame shift or large deletions within the protein, or splice site mutations, resulting in altered splice products of mRNA.

A comprehensive diagnostic work-up was performed in all centers including medical history and cardiac, renal, and neurological evaluation. Data documentation followed the clinical practice of the interdisciplinary Fabry Expert Centers. A detailed clinical work-up of patients was reported previously [10]. Gastrointestinal symptoms include abdominal pain, tenesmus, or cramping more than once a week. Diarrhea was defined as ≥ 1 day/ month with three loose bowels or > 250 g of stool weight per day. Fatigue was defined by the Fukuda criteria [11]. Cardiac assessment included echocardiography with left ventricular hypertrophy (LVH) defined as an interventricular septum thickness in diastole (IVSd) ≥ 11.5 mm, which is the same definition of LVH as used for the Disease Severity Scoring System (DS3) [12].

Renal function was quantified by estimated glomerular filtration rate (eGFR) using the Chronic Kidney Disease-Epidemiology Collaboration equation (CKD-EPI) based on serum creatinine [13] and the albumin-to-creatinine ratio (ACR) from spot urine. Renal impairment was defined as an eGFR < 90 ml/min/1.73 m^2^ according to European FD guidelines [4] and albuminuria as ACR > 30 mg albumin per g creatinine. Patients underwent neurological examination and a clinical interview focusing on a history of cerebral stroke or TIAs. Additionally, the presence of FD-related pain was investigated [14]. Disease severity was assessed using the DS3 [12] and the Mainz Severity Score Index (MSSI) [15]. Additional concomitant medication was assessed for every visit. Renin-angiotensin-aldosterone-system (RAAS) blockers include the prescription of angiotensin-converting enzyme blockers, angiotensin receptor blockers, renin blockers, as well as aldosterone antagonists. Diuretics include the prescription of high ceiling/loop diuretics, thiazides, carbonic anhydrase inhibitors, and potassium-sparing diuretics. Analgesic drugs include the prescription of opioids, anticonvulsants, selective serotonin reuptake inhibitors, and non-steroidal anti-inflammatory drugs.

Plasma lyso-Gb_3_ levels were measured at Centogene (Rostock, Germany) or Archimed Life Science GmbH (Vienna, Austria) using LC-MS/MS with a lower limit of detection of < 0.7 ng/ml and an upper limit of normal < 1.9 ng/ml.

### Statistical analysis

If not stated otherwise, continuous variables are expressed as median with range, since most data were unequally distributed. Categorical data are expressed as numbers and relative frequencies in percent. A quality control of assessed data is provided in the supplements (Supplemental Table 3) showing an overall data completeness of 90.6%. To deal with missing data, analyses were performed with an as-is state for every parameter with individual patient numbers provided within tables and figures. Differences in a group between the three visits (T1, T2, and T3) were analyzed with a Friedman test and differences between groups were analyzed with the Kruskal-Wallis test for continuous data. A Chi-square test was used for categorical data. Fisher’s exact test was used to calculate the relative risk (RR) for concomitant medications between T1 and T3. Mean changes for IVSd, eGFR, lyso-Gb_3_, DS3, and MSSI were individually calculated based on three consecutive values from T1 to T3 and significant changes were analyzed using the Wilcoxon Signed Rank test. Statistical significance was considered at a 2-sided *p* < 0.05. SAS version 9.4 (SAS Institute Inc., Cary, North Carolina, USA) and GraphPad PRISM V8.4 software (GraphPad Software Inc., La Jolla, CA, USA) were used for statistical analyses and data visualization.

## Results

### Clinical characterization of female patients with Fabry disease

To assess the organ damage and impact of ERT (including treatment with agalsidase-alfa and agalsidase-beta), 159 female patients with genetically confirmed FD were recruited at 6 FD centers (Fig. 1A). Patients’ data were assessed from 3 consecutive visits T1, T2, and T3 (Fig. 1B). According to their FD-specific treatment, patients were separated in 3 groups: (i) untreated (ERT-naïve) group, with patients never treated with ERT before as well as between T1 and T3 (*n* = 71), (ii) newly ERT-treated group, with patients naïve to any ERT before T1, but ERT-treatment (with either agalsidase-alfa or -beta) directly initiated after T1 (*n* = 47), (iii) long-term ERT-treated group with patients continuously treated with agalsidase-alfa or -beta before T1 (median duration of 89 [7 to 189] months before T1) and also between T1 and T3 (*n* = 41; Fig. 1B). The median observational time between T1 and T3 were 62 [22 to 192] months, 66 [24 to 170] months, and 49 [22 to 183] months for the untreated group, the newly ERT-treated group and the long-term ERT-treated group, respectively (Fig. 1B) and did not differ significantly between the groups (*p* = 0.2934). The distribution of patients receiving agalsidase-alfa or -beta in newly-treated and long-term ERT-treated patients differed slightly (Fig. 1C; *p* = 0.0342). In detail, the frequency of agalsidase-beta treated patients increased from 17 to 38% between long-term and newly treated patients (Fig. 1C).

Baseline characteristics of the study cohort at T1 are provided in Table 1. In detail, female patients within the untreated (ERT-naïve) group (*n* = 71) were younger (*p* = 0.0004), presented with the lowest plasma lyso-Gb_3_ values (*p* = 0.0001) and the lowest frequencies of typical FD-related symptoms and manifestations, including edema, angiokeratoma, and fatigue (Table 1). They also suffered less often from FD-related pain and presented with the lowest IVSd values (*p* = 0.0001), resulting in a lower risk for LVH (*p* = 0.0002; Table 1). Overall, a less severe disease load in the untreated patients was also reflected by lowest DS3 and MSSI scores (both *p* = 0.0001, respectively; Table 1). Of note, patients within the newly ERT-treated group (*n* = 47) presented with the highest blood pressure values, resulting in an increased risk for hypertension (*p* = 0.0250), showed more frequent albuminuria (*p* = 0.0244) and presented with the highest frequencies of nonsense *GLA* mutations (*p* = 0.0033; Table 1). The median eGFR values and frequency of TIAs/strokes before T1 did not differ significantly between all three groups (Table 1). An analysis of concomitant medications showed that ERT-treated patients (long-term as well as newly treated) were more often treated with RAAS blockers and antidepressants at T1 (Table 1).

**Table 1:** Baseline characteristics of the study cohort

	*n*	newly ERT-treated	*n*	long-term ERT-treated	*n*	untreated (ERT-naïve)	*p*-value
age, years	47	49 [25 to 73]*	41	48 [16 to 75]*	71	34 [18 to 72]	**0.0004**
BMI, kg/m²	46	27.7 [18.8 to 35.5]	40	23.9 [18.3 to 38.3]	69	23.1 [9.9 to 48.5]	0.1980
SBP, mmHg	45	135 [99 to 199]	39	119 [100 to 152]^#^	69	118 [87 to 160]^#^	**0.0002**
DBP, mmHg	45	80 [52 to176]	39	70 [56 to 100]^#^	69	75 [55 to 98]^#^	**0.0007**
nonsense mutations, *n*	47	30 (63.8)	41	20 (48.8)	71	23 (32.4)	**0.0033**
hypertension^a^, *n*	45	13 (26.7)*	39	4 (10.3)^#^	69	8 (11.6)	**0.0250**
plasma lyso-Gb_3_, ng/ml	31	9.5 [3.4 to 19.3]*	31	8.1 [2.5 to 14.5]*	63	3.1 [0.2 to 15.8]	**0.0001**
plasma lyso-Gb_3_ > reference, *n*	31	31 (100.0)	31	31 (100.0)	63	38 (60.3)	**0.0001**
edema, *n*	47	3 (6.4)	41	7 (17.1)	67	1 (1.5)	**0.0090**
GI symptoms, *n*	42	11 (26.2)	21	4 (19.0)	59	22 (37.3)	0.2279
angiokeratoma, *n*	47	17 (36.2)	41	20 (48.8)	66	16 (24.2)	**0.0327**
cornea verticillata, *n*	32	17 (53.1)	20	16 (80.0)	34	13 (38.2)	**0.0121**
tinnitus, *n*	41	8 (19.5)	21	5 (23.8)	58	3 (5.2)	**0.0352**
hypoacusis, *n*	42	4 (9.5)	21	4 (19.0)	58	3 (5.2)	0.1810
fatigue, *n*	38	7 (18.4)	21	6 (28.6)	56	3 (5.4)	**0.0199**
FD-related pain, *n*	47	30 (63.8)	41	30 (73.2)	71	28 (39.4)	**0.0010**
TIA/stroke, *n*	47	5 (10.6)	40	9 (22.5)	71	6 (8.5)	0.1893
DS3 total score	42	10 [1 to 26]*	39	9 [1 to 26]*	61	3 [0 to 19]	**0.0001**
MSSI total score	45	12 [2 to 29]*	40	17 [3 to 37]*	63	1 [0 to 7]	**0.0001**
IVSd, mm	40	11.0 [8.0 to 26.0]*	35	11.0 [7.0 to 21.0]*	62	8.5 [5.0 to 16.0]	**0.0001**
LVH, *n*	40	19 (46.3)	35	15 (42.9)	62	8 (12.9)	**0.0002**
pacemaker/ICD, *n*	44	2 (4.5)	41	1 (2.4)	71	0 (0.0)	0.2172
serum creatinine, mg/dl	47	0.72 [0.46 to 1.84]	39	0.72 [0.49 to 1.24]	68	0.74 [0.51 to 1.28]	0.6077
eGFR, ml/min/ 1.73 m²	47	96 [33 to 135]	39	97 [43 to 142]	68	101 [43 to 134]	0.3356
CKD G1	47	29 (61.7)	39	28 (71.8)	68	48 (70.6)	0.5154
CKD G2	47	17 (36.2)	39	10 (25.6)	68	19 (27.9)	0.5106
CKD G3	47	1 (2.1)	39	1 (2.6)	68	1 (1.5)	0.9201
ACR, mg/g protein	36	50 [0 to 2436]*	38	26 [0 to 1443]	53	19 [0 to 201]	**0.0024**
albuminuria, *n*	36	24 (66.7)	38	17 (44.7)	53	20 (37.7)	**0.0244**
dialysis, *n*	47	1 (2.1)	41	0 (0.0)	71	0 (0.0)	0.3015
KTX, *n*	47	1 (2.1)	41	0 (0.0)	71	0 (0.0)	0.3015
RAAS blocker, *n*	42	16 (38.1)	39	15 (38.5)	65	9 (13.8)	**0.0045**
diuretics, *n*	43	5 (11.6)	39	4 (10.3)	66	1 (1.5)	0.0722
analgesics, *n*	40	4 (10.0)	39	5 (12.8)	66	7 (10.6)	0.9127
antidepressants, *n*	35	6 (17.1)	20	5 (25.0)	56	2 (3.6)	**0.0183**
≥ 3 organ manifestations justifying ERT, *n*	47	11 (23.3)	41	7 (17.1)	71	5 (7.0)	**0.0403**

Albuminuria was defined as an albumin/creatinine ratio (ACR) > 30 mg albumin per gram of creatinine from spot urine. Nonsense mutations include the presence of nonsense mutations, insertions/deletions and intronic mutations. Continuous variables are expressed as median with range, since most data were unequally distributed. Categorical data are expressed as numbers and relative frequencies in percent. BMI: body mass index, CKD: chronic kidney disease, with CKD G1 ≥ 90 ml/min/1.73 m², CKD G2: 60–89 ml/min/1.73 m² and CKD G3: 30–59 ml/min/1.73 m², DBP: diastolic blood pressure, DS3: disease severity scoring system, eGFR: estimated glomerular filtration rate, ERT: enzyme replacement therapy (includes treatment with agalsidase-alfa or -beta). FD: Fabry disease, GI: gastrointestinal symptoms including the presence of diarrhea and/or abdominal pain, ICD: implantable cardioverter device, IVSd: interventricular septum thickness in diastole, KTX: kidney transplantation, lyso-Gb_3_: globotriaosylsphingosine with an upper limit of normal of 1.8 ng/ml, LVH: left ventricular hypertrophy, defined as IVSd > 11.5 mm, MSSI: Mainz severity score index, RAAS: renin-angiotensin-aldosterone system, SBP: systolic blood pressure, TIA: transient ischemic attack. a: hypertension without target blood pressure value achievement (120–130/70–80 mmHg); *p < 0.01 versus untreated (ERT-naïve), ^#^*p* < 0.01 versus newly treated

### Implementation of FD guidelines for the treatment of females

**Fig. 2:**
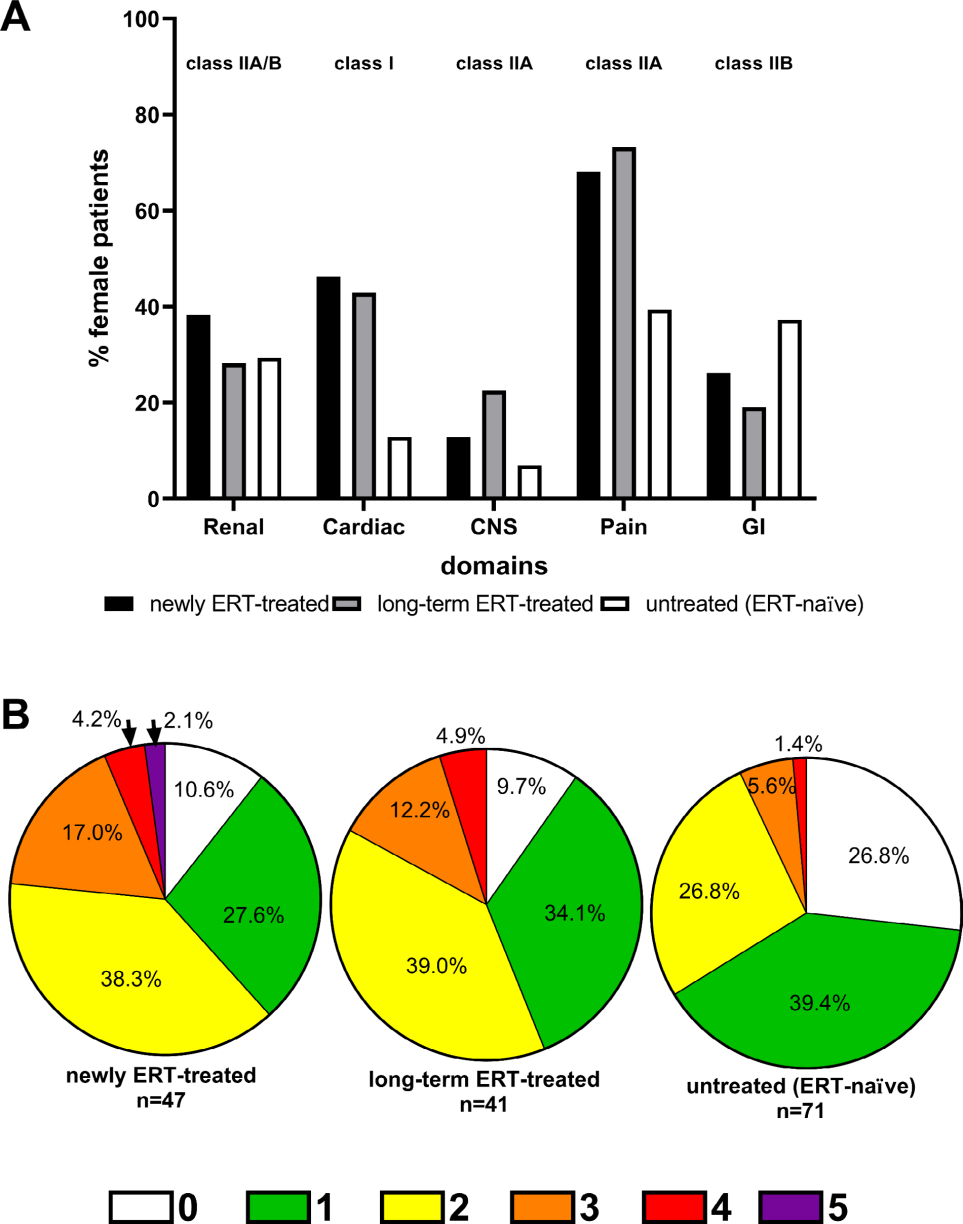
Differences between the three analyzed patient groups presenting with organ manifestation justifying treatment with enzyme replacement therapy (ERT) according to current guidelines at baseline (T1). (**A**) Distribution of manifestations in different organs justifying ERT with class I (ERT is recommended) and class IIA/B (ERT should/may be considered) recommendations. (**B**) Accumulation of different organ manifestations justifying ERT. Females may present with zero to five different manifestations (white, green, yellow, orange, red, purple represent 0, 1, 2, 3, 4, 5 simultaneous manifestations, respectively). Cardiac: left ventricular hypertrophy, Renal: eGFR < 90 ml/min/1.73 m^2^, CNS: stroke/ transient ischemic attack, Pain: neuropathic/FD-related pain, GI: gastrointestinal symptoms (diarrhea, abdominal pain)

Next, we analyzed whether patients were treated in accordance with current European FD guidelines [4] (class I: ERT is recommended; class IIA/B: ERT should/may be considered) at the onset of first organ manifestations at T1. Comparable to our previous study [10], we analyzed the presence of at least one of the following manifestations in different organs (cardiac: LVH, renal: eGFR < 90 ml/min/1.73 m^2^, CNS: stroke/TIA, pain: FD-related neuropathic pain, gastrointestinal: gastrointestinal symptoms) justifying ERT initiation according to classes I to IIA/B recommendations [4] (Fig. 2). Following this stratification, we identified FD-related pain as most frequent in all three groups, followed by cardiac and renal involvement (Fig. 2A). Of note, untreated patients reported gastrointestinal symptoms very often (37.3%; Fig. 2A). Next, we assessed the cumulative manifestations of organ damages and symptoms, revealing that only about 10% of ERT-treated patients neither showed increased IVSds or decreased eGFRs, nor suffered from TIAs/strokes, FD-related pain or gastrointestinal symptoms (Fig. 2B). Importantly, most ERT-treated patients presented with 2 or more cumulative manifestations (Fig. 2B). 26.8% of untreated patients showed no organ damage or manifestation at T1 and only one third of all untreated females presented with more than 1 manifestation (Fig. 2B). A detailed overview of the individual combinations of organ involvements justifying treatment with ERT according to current guidelines is presented in Supplemental Table 4.

### Clinical courses of the three groups

Next, we analyzed the clinical course of the three groups over time. The frequencies of transient ischemic attacks and strokes are presented in Table 2. During T1 and T3 five events occurred in newly ERT-treated patients, three events occurred in long-term ERT treated patients, two events occurred in untreated (ERT-naïve) patients, resulting in frequencies of 16.6 events /1,000 person years, 13.8 events /1,000 person years and 4.9 events /1,000 person years, respectively (Table 2). Of note, in newly ERT- treated patients, four of these events occurred in patients previously unaffected by any cerebrovascular event. In long-term ERT-treated and untreated patients one event occurred in a patient previously unaffected by any cerebrovascular event, respectively (Table 2).

**Table 2:** Frequencies of transient ischemic attacks and strokes

	patients with events before T1, *n* (%)	events between T1 and T3, *n* (%)	patients with de novo events, *n* (%)	total observation time between T1 and T3 (years)	frequency (events/1.000 patient years)
newly ERT-treated [*n* = 47]	5 (10.6)	5 (10.6)	4 (80.0)	301	16.6
long-term ERT-treated [*n* = 40]	9 (22.5)	3 (7.5)	1 (33.3)	217	13.8
untreated [*n* = 71]	6 (8.5)	2 (3.0)	1 (50.0)	406	4.9

Patients with de novo events are defined as patients previously unaffected by any cerebrovascular event. ERT: enzyme replacement therapy (includes treatment with agalsidase-alfa or -beta)

**Fig. 3:**
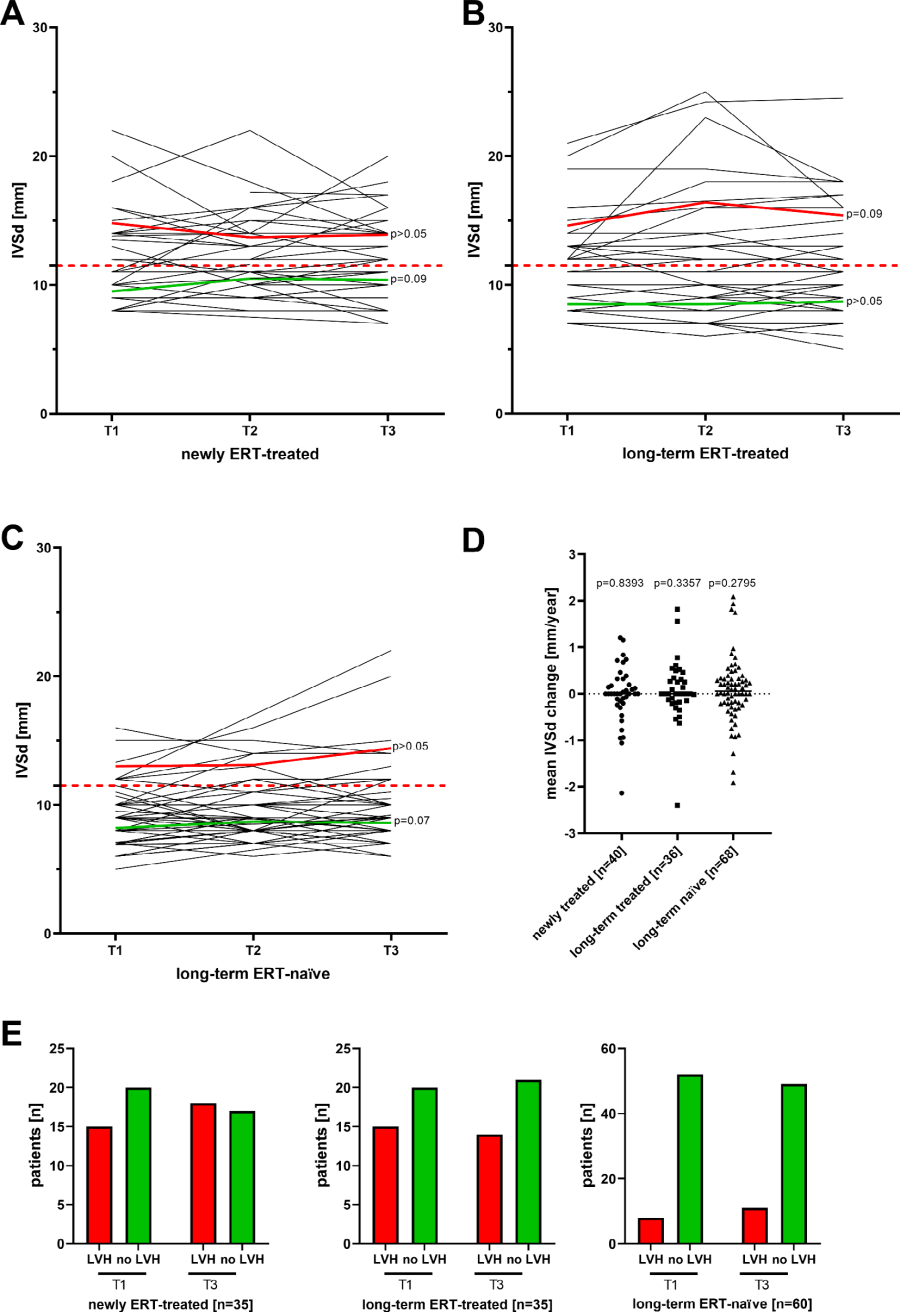
Changes of left ventricular septum thickness over time. (**A**) to (**C**) Individual left ventricular septum thickness in diastole (IVSd) in the three groups at the three visits (T1, T2 and T3). The red dotted lines at 11.5 mm mark the cut-off value for the presence of left ventricular hypertrophy (LVH). The red and green solid lines mark the mean values for patients with and without LVH (at T1), respectively. (**D**) Yearly IVSd change within the three groups. (**E**) Number of patients with (red bars) and without (green bars) LVH (defined as an IVSd > 11.5 mm, red bars) at T1 and T3 in the three groups. ERT: Enzyme replacement therapy includes the treatment with either agalsidase-alfa or -beta

To assess the cardiac involvement over time, we analyzed the IVSd as a marker for LVH (Fig. 3A-D). Overall, mean IVSd changed not significantly between T1 and T3 and, thus, was stable in all three groups (newly ERT-treated group: -0.01 ± 0.61 mm/year; *p* = 0.8393; long-term ERT-treated group: 0.07 ± 0.65 mm/year, *p* = 0.3357; untreated group: 0.08 ± 0.70 mm/year, *p* = 0.2795; Fig. 3D). In addition, there was no significant effect of the presence of LVH at T1 on IVSd outcome, although long-term ERT-treated patients with LVH at T1 tended to show a slight but not significant increase of IVSd despite treatment (*p* = 0.09; Fig. 3B). Furthermore, the frequencies of LVH within the three groups between T1 and T3 were stable (newly ERT-treated: *p* = 0.6324; long-term ERT-treated group: *p* = 0.9999; untreated group: *p* = 0.6179; Fig. 3E). Since life-threatening arrhythmia are a major cause for sudden death in FD, the implementation of ICDs and pacemakers was assessed over time. In untreated females no (0.0%) patient required an ICD or pacemaker during the observational time. In newly and long-term ERT-treated patients, 4 (8.5%) and 2 (4.9%) patients required an ICD/pacemaker between T1 and T3, respectively. For completeness, no myocardial infarctions were detected in any of the three groups, neither before T1 nor between T1 and T3.

**Fig. 4:**
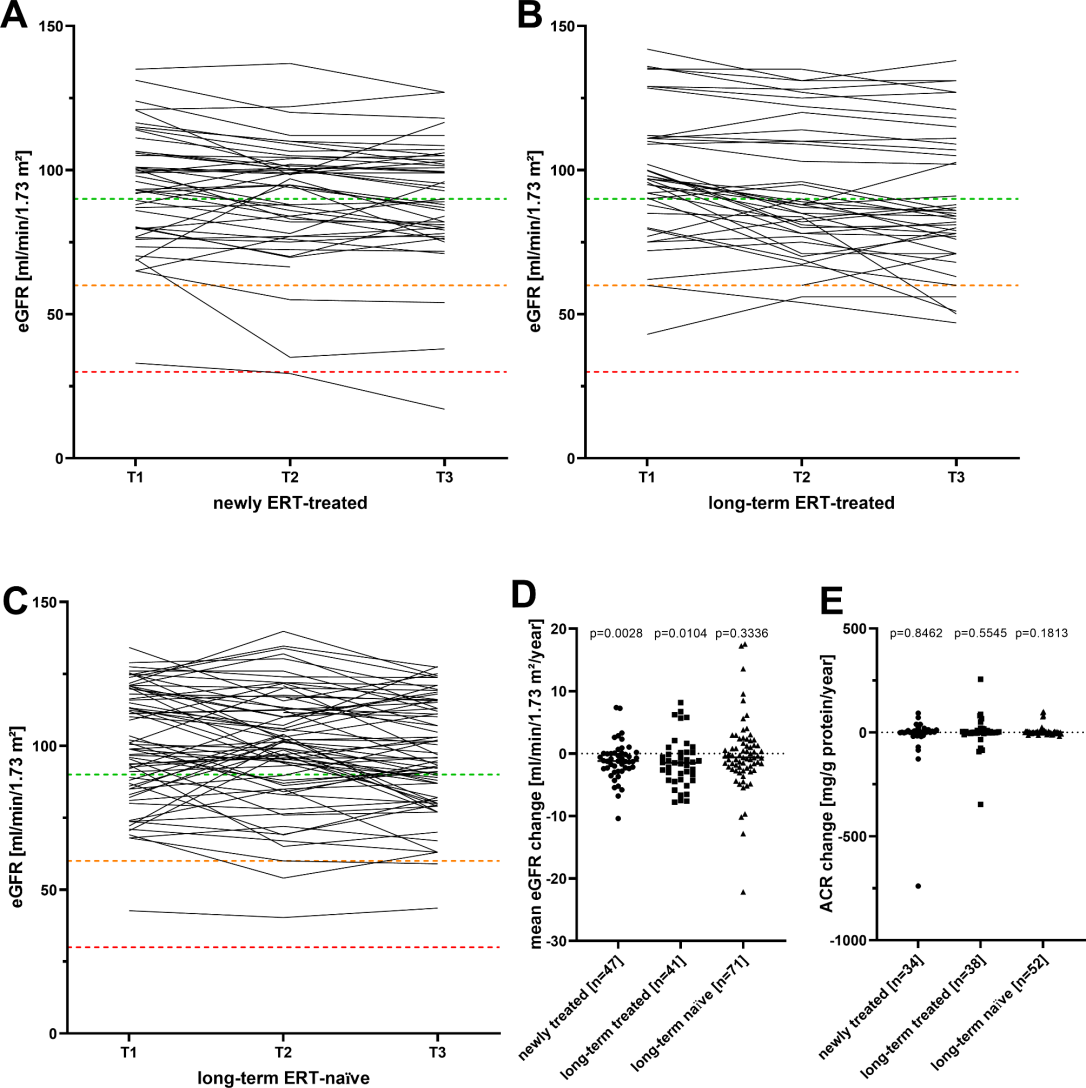
Changes in kidney function over time. (**A**) to (**C**) Individual estimated glomerular filtration rate (eGFR) values in the three groups at the three visits (T1, T2 and T3). The dotted lines mark the cut-off values for CKD G1 (green), CKD G2 (orange) and CKD G3 (red). (**D**) Annualized eGFR changes within the three groups. (**E**) Yearly albumin/creatinine ratio (ACR) changes within the three groups. ERT: Enzyme replacement therapy includes the treatment with either agalsidase-alfa or -beta

As a marker for renal involvement, CKD-EPI-based eGFR and ACR values were analyzed between T1 and T3. Mean eGFRs in newly and long-term ERT-treated patients slightly decreased (newly ERT-treated: -1.1 ± 3.1 ml/min/1.73 m^2^/year, *p* = 0.0028; long-term ERT-treated group: -1.4 ± 3.9 ml/min/1.73 m^2^/year, *p* = 0.0104) and remained stable within the untreated group: -0.2 ± 5.6 ml/min/1.73 m^2^/year, *p* = 0.3336; Fig. 4A-D). ACR values were stable in all three groups over time (newly ERT-treated: 0.3 [-740 to 93] mg/g/year, *p* = 0.8462; long-term ERT-treated group: 0.0 [-346 to 255] mg/g/year, *p* = 0.5545; untreated group: 0.7 [-19 to 100] mg/g/year, *p* = 0.1813; Fig. 4E).

Since IVSd, eGFR as well as ACR values can be influenced by cardio-/renoprotective medication (RAAS blockers) and diuretics, we also analyzed potential changes in the prescription of concomitant medications between T1 and T3 (Table 3). At the end of observation at T3 only newly ERT-treated patients received RAAS-blockers more often (1.55 [CI 95% 1.00 to 2.48]), while use of these drugs in long-term ERT-treated and untreated patients was stable (Table 3). The prescription of diuretics, analgesics and, antidepressants was stable in all groups (Table 3).

**Table 3:** Changes in concomitant medication

	treated at T1, *n* (%)	changes, *n*	treated at end of observation (T3), *n* (%)	relative risk [95% CI]
**RAAS blocker**				
newly ERT-treated	16 (38.1)	+ 12/-3	25 (59.5)	1.55 [1.00 to 2.48]
long-term ERT-treated	15 (38.5)	+ 5/-2	18 (46.2)	1.17 [0.75 to 1.91]
untreated	9 (13.8)	+ 5/-2	12 (18.5)	1.20 [0.77 to 2.15]
**diuretics**				
newly ERT-treated	5 (11.6)	+ 3/-2	6 (14.0)	1.11 [0.65 to 2.45]
long-term ERT-treated	4 (10.3)	+ 6/-1	9 (23.1)	1.75 [0.88 to 4.35]
untreated	1 (1.5)	+ 1/-0	2 (3.0)	1.51 [0.61 to 8.24]
**analgesics**				
newly ERT-treated	4 (10.0)	+ 4/-1	7 (17.5)	1.43 [0.76 to 3.52]
long-term ERT-treated	5 (12.8)	+ 4/-1	8 (20.5)	1.36 [0.75 to 3.04]
untreated	7 (10.6)	+ 2/-3	6 (9.1)	0.92 [0.61 to 1.74]
**antidepressants**				
newly ERT-treated	6 (17.1)	+ 1/-4	3 (8.6)	0.71 [0.46 to 1.40]
long-term ERT-treated	5 (25.0)	+ 1/-1	5 (25.0)	1.00 [0.54 to 2.25]
untreated	2 (3.6)	+ 2/-0	4 (7.1)	1.53 [0.70 to 5.32]

ERT: enzyme replacement therapy (includes treatment with agalsidase-alfa or -beta), RAAS: renin-angiotensin-aldosterone system

### Disease load and biochemical response over time

**Fig. 5:**
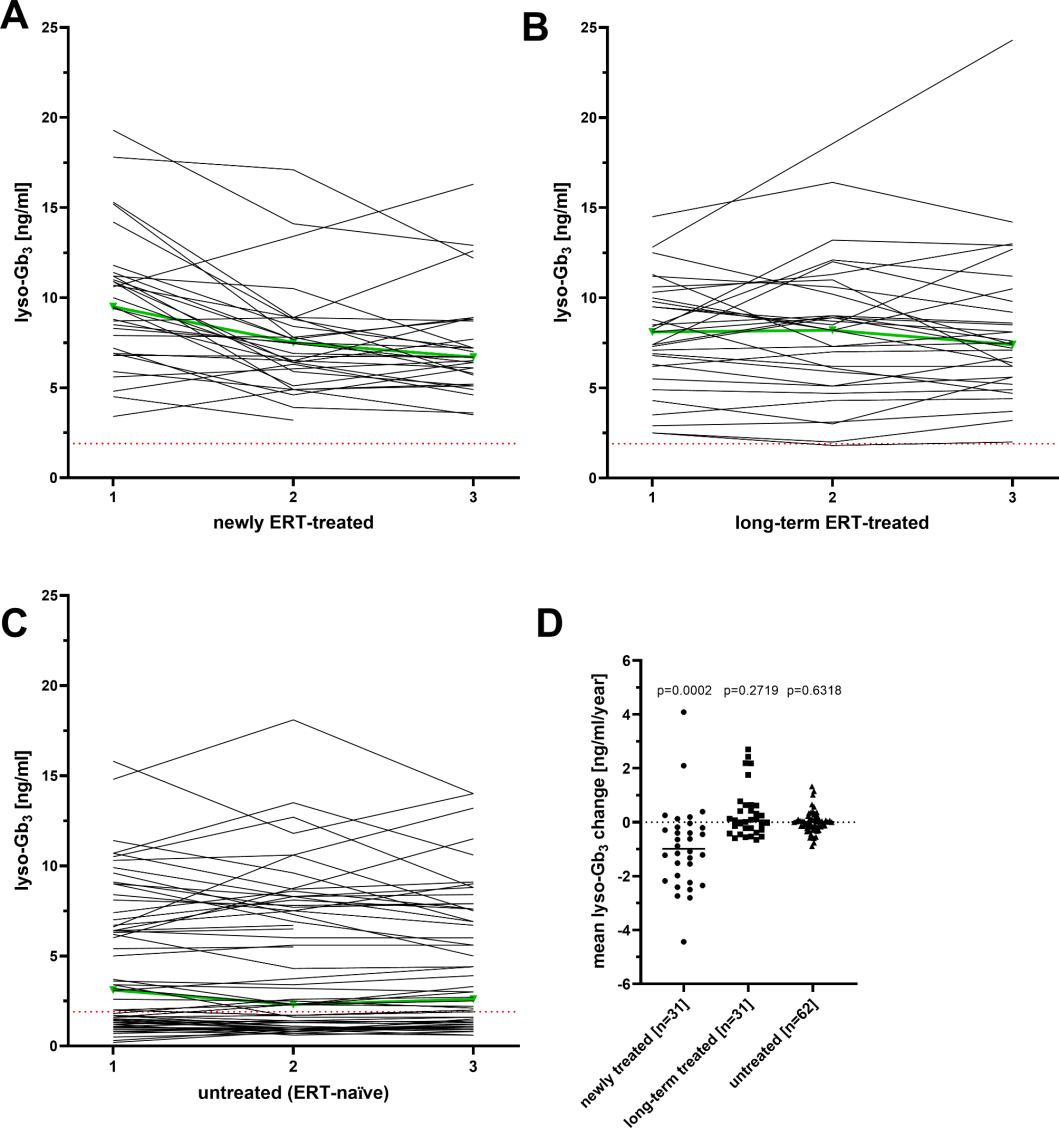
Changes in plasma lyso-Gb_3_ over time. (**A**) to (**C**) Individual plasma lyso-Gb_3_ values in the three groups at the three visits (T1, T2 and T3). The red dotted line marks the upper limit of normal at 1.9 ng/ml. Bright green lines and triangles represent the median values of the respective groups. (**D**) Yearly lyso-Gb_3_ changes within the three groups. ERT: Enzyme replacement therapy includes the treatment with either agalsidase-alfa or -beta. lyso-Gb_3_: globotriaosylsphingosine

To assess a change of the disease load, total DS3 and MSSI scores were analyzed over time. Independent of the analyzed group, both scores slightly increased over time, as follows: DS3: newly ERT-treated: 0.2 [-4.2 to 4.1] /year, *p* = 0.0474; long-term ERT-treated group: 0.2 [-2.8 to 4.5] /year, *p* = 0.0142; untreated group: 0.2 [-3.5 to 7.9] /year, *p* = 0.0124; Supplemental Fig. 1). MSSI: newly ERT-treated: 1.0 [-5.7 to 3.8] score/year, *p* = 0.0043; long-term ERT-treated group: 0.4 [-2.9 to 7.2] score/year, *p* = 0.0014; untreated group: 0.1 [-0.3 to 4.0] score/year, *p* = 0.0536; Supplemental Fig. 1). As a marker for the biochemical response especially in ERT-treated patients, plasma lyso-Gb_3_ was measured over time (Fig. 5). Plasma lyso-Gb_3_ values in the newly ERT-treated group decreased significantly over time (-0.95 [-4.44 to 4.08] ng/ml/year, *p* = 0.0002) and remained stable in both other groups (long-term ERT-treated group: 0.06 [-0.65 to 2.70] ng/ml/year; *p* = 0.2719; untreated group: 0.00 [-0.89 to 1.33] ng/ml/year; *p* = 0.6518; Fig. 5D).

## Discussion

The aim of our multicenter study was to investigate the implementation of the European FD guidelines and recommendations in female patients with FD and to analyze the impact of agalsidase-alfa and agalsidase-beta in newly and long-term ERT-treated females. Furthermore, we analyzed the disease course in untreated females over time.

Our main findings are: 1) ∼ 90% of female FD patients treated with ERT presented with at least one organ manifestation justifying treatment according to current European guidelines [4]; 2) untreated females showed a less severe disease load with less FD-typical organ damage; 3) newly and long-term ERT-treated patients as well as untreated females presented with a stable septal thickness; 4) ERT-treated females presented with a slight decrease of renal function over time; 5) plasma lyso-Gb_3_ levels were higher in ERT-treated females and decreased in those who were newly ERT-treated.

### Implementation of FD guidelines at T1

By contrast to male FD patients, the optimal time point for ERT initiation in females with FD, often still classified as “only” asymptomatic or minor symptomatic patients, remains unclear, as their disease manifestations and progression, as well as biomarker levels are diverse. Current guidelines recommend ERT initiation in females with FD as soon as FD-typical organ manifestations such as LVH, renal insufficiency, or clinical events including TIA or stroke appear [4, 5]. In our cohort, nearly all patients who received ERT prior to T1 (long-term ERT-treated) or started ERT after T1 (newly ERT-treated), showed at least one organ manifestation defined as LVH, an eGFR < 90 ml/min/1.73 m^2^, cerebral complications (stroke/TIA), FD-related pain, or gastrointestinal symptoms justifying ERT. Most of these patients presented with 2 or more of these manifestations. Nevertheless, 73.2% of the untreated (ERT-naïve) patients also presented with one or more manifestations. Whether these manifestations are all FD-related, and if the affected patients present with disease progression over time due to absent ERT needs clarification. At baseline, untreated patients were less severely affected compared to ERT-treated patients (newly- as well as long-term ERT-treated). This lower disease load is reflected by lower plasma lyso-Gb_3_ levels, a lower risk for FD-related symptoms (such as tinnitus and fatigue), and less cardiac involvement resulting finally in lower disease scores. The relatively high incidence of gastrointestinal symptoms might be explained by the fact that these symptoms are difficult to record and are often very subjective. Stroke and TIAs in the young is a common manifestation of FD, even in affected females. According to a review by Mehta and Ginsberg [16], the overall frequency of strokes/TIAs in untreated females is ∼ 16.7%. In our cohort, at T1 the frequencies in ERT-treated patients (newly and long-term) were 10.5% and 22.5%, respectively, and, thus, within the expected range. In untreated ERT-naïve females, the frequency for such cerebrovascular events was only 8.5% at T1, also pointing towards a lower disease load in these patients.

However, since untreated females were also significantly younger at T1, the lower disease load might still be explained by age, which could be addressed by our subsequent longitudinal analyses.

In untreated Fabry patients, there are various reasons for the lack of Fabry-specific therapy: (1) The organ manifestations are most likely due to untreated or poorly treated comorbidities (e.g., hypertension). (2) Patients refuse treatment even though they have been fully informed. (3) Patients do not want to start treatment if they are planning a pregnancy due to the product information for agalsidase-alfa and -beta. (4) A lack of insurance status can be ruled out as a reason for non-treatment in our cohort.

Quality of life is often decreased in female patients with FD. Concerning the initiation of FD-specific therapy in females, the current guidelines are based exclusively on measurable organic manifestations and additionally only consider refractory pain [3–5]. Certainly, the limited quality of life in females with FD should be given greater consideration.

### Disease progression in ERT-treated and untreated females

A recent review reported that female patients under ERT demonstrate cardiac stabilization over time [2]. Indeed, our data of newly as well as long-term ERT-treated females confirmed stable values for septum thickness independent of the presence of LVH at T1. Interestingly, also the untreated females did not show any change in IVSd.

Progressive loss of renal function increases morbidity and mortality in FD. Depending on the stage of kidney disease, affected female patients can show loss of eGFR of up to 3 ml/min/1.73 m²/year, if untreated [17]. The effects of FD-specific treatment on eGFR in females are heterogeneous and are affected by mutation, age, CKD stage, albuminuria, and comorbidities (such as hypertension) [2]. Furthermore, the natural decline of eGFR by ∼ 1 ml/min/1.73 m²/year starting in the third decade of life [18] needs to be taken into account, when analyzing renal function over time. In our cohort, the females from both ERT-treated groups showed a yearly decline of 1.1 to 1.4 ml/min/1.73 m^2^/year and remained stable within the untreated group. This means that the treated women were stable and only show the natural eGFR decline typical for this age. Since the untreated women were much younger (34 years versus ∼ 49 years), no age-related eGFR decline was observed.

Concomitant medication with RAAS blockers and diuretics is an important aspect for FD patients concerning renal and cardiac protection and antihypertensive effects. Due to the decrease in glomerular pressure, these agents must be taken into account when assessing the yearly eGFR decline. In our cohort, prescription of concomitant medication did not change significantly. Only newly ERT-treated patients presented with an increase of RAAS blockers over time. This is best explained by the guideline-based initiation of RAAS blockers in newly diagnosed albuminuria, eGFR decrease and LVH. The aim of RAAS blockage is a long-term therapeutic stabilization of renal function; a functionally-related decrease in eGFR shortly after the start of therapy was not recorded. Thus, a significant effect of concomitant medication on eGFR can be excluded in our study. The disease scores based on MSSI and DS3 slightly increased over time in all three groups. This might lead to the impression that the patients show at least some disease progression. However, many aspects which are queried in both scores are linked to age (such as renal function). Thus, even in stable patients, disease scores can slightly increase over time. A more suitable tool to assess early changes in disease progression might be the FAbry STabilization indEX (FASTEX) tool, which aims to quantify the clinical stability or disease progression between two consecutive evaluations [19]. However, since FASTEX is not used in the clinical routine of the participating FD centers, this tool was not used in this study. Edema, fatigue, depression, and also tinnitus are typical FD-related symptoms and manifestations. Interestingly, we did not find any significant differences in these symptoms and manifestations between newly ERT-treated and long-term ERT-treated patients. Significant differences were only found in comparison to untreated patients, indicating a lower disease burden in these patients, requiring less concomitant medication including antidepressants or diuretics, as well. However, it is possible that the long-term treated patients were more often under calcium antagonists with the known side effect profile of ankle edema. However, as we only have incomplete data concerning antihypertensive comedication, this can only be an assumption. Since short-term and long-term treated patients presented with comparable disease conditions at baseline at the same age, it could be hypothesized that the long-term treatment with ERT at least resulted in a disease stabilization.

Plasma lyso-Gb_3_ values are generally lower in females than in males [20]. Nevertheless, there is a reduction in lyso-Gb_3_ during ERT [2]. In our cohort, only newly ERT-treated females presented with a significant decrease of plasma lyso-Gb_3_ levels, while both other groups were stable. This can be explained by the fact that lyso-Gb_3_ rapidly reaches a maximum after childhood [21] and under FD-specific treatment, plasma lyso-Gb_3_ levels will decrease to a plateau after ∼ 3 months of therapy initiation [22]. This level was reached in the long-term ERT-treated group before T1. Of note, plasma lyso-Gb_3_ plateaus vary between individuals and are dependent on initial lyso-Gb_3_ levels, the underlying mutation and the ERT dose [23].

In clinical care, the comparison of individual treatment strategies is of great interest. When comparing patients treated with agalsidase-alfa and agalsidase-beta in our cohort, there was no significant difference in the clinical course (eGFR; cardiac septal thickness; renal, cardiac, and neurological events). A detailed presentation of these results is the aim of a further study.

We conclude, both severely affected females who were treated and less severely affected untreated females showed a broadly stable disease course over 5 years. The treatment decisions based on the European guidelines appear to have been correct in most patients of our cohort. Untreated females with organ manifestations must be evaluated with regard to the cause of their organ involvement, as this determines the correct decision to initiate FD-specific treatment. Individually tailored treatment decisions based on European FD guidelines to initiate ERT appear to be a suitable option for disease stabilization, at least in our cohort of ERT-treated females.

## Limitations

Due to the COVID19 pandemic the number of total recruited female patients with long-term follow-up data was limited. Due to the retrospective approach, magnetic resonance imaging (MRI) data for cardiac and cerebral manifestations were limited and, thus, not evaluated. Only IVSd rather than left ventricular mass indices data were sufficiently available for all patients. Furthermore, due to this approach, patients with asymmetric left ventricular hypertrophy (e.g. apical change only) were excluded, which is a limitation. Since only living patients were recruited at T3, no conclusions can be drawn concerning FD-related mortality. Due to the study design of this investigator-initiated study, patients receiving migalastat were not included in this study. Regarding the implementation of treatment guidelines, this is a limitation since not all treatment options (migalastat) were covered. No data concerning quality of life were assessed in this study, which is a limitation. Further studies are warranted to analyze potential effects of different FD-specific treatment options on quality of life.

## Electronic supplementary material

Below is the link to the electronic supplementary material.

Supplementary Material 1

## Data Availability

The original contributions presented in the study are included in the article/Supplementary Material, further inquiries can be directed to the corresponding author.

## Abbreviations

AGAL/GLA: α-galactosidase A
ACR: Albumin-to-creatinine ratio
CKD-EPI: Chronic Kidney Disease-Epidemiology Collaboration equation
DS3: Disease Severity Scoring System
eGFR: Estimated glomerular filtration rate
ERT: Enzyme replacement therapy
FD: Fabry disease
FASTEX: FAbry STabilization indEX
Gb_3_: Globotriaoslyceramide
IVSd: Interventricular septum thickness in diastole
lyso-Gb_3_: Globotriaosylsphingosine
LVH: Left ventricular hypertrophy
MRI: Magnetic resonance imaging
MSSI: Mainz Severity Score Index
RAAS: Renin-angiotensin-aldosterone-system
RR: Relative risk
TIA: Transient ischemic attack
