# Supplementary material for: Impact of enzyme replacement therapy on clinical manifestations in females with Fabry disease
Source: Orphanet J Rare Dis. 2024 Dec 27;19:490. doi: 10.1186/s13023-024-03503-4 (PMC11673826; doi:10.1186/s13023-024-03503-4)
Supplement: Supplementary file 1 — Supplementary Material 1 [file 13023_2024_3503_MOESM1_ESM.docx]

| **Supplemental Table 1: Recommendations for initiation of ERT in adult male and female patients with classic or later-onset mutations, or GLA variance of unknown significance according to Ortiz et al. *Mol Genet Metab*. 2018;123:416-427.** | |
| --- | --- |
| Adult patient population | Recommendation for the initiation of ERT |
| Classic Fabry mutation:  Male patient, symptomatic or asymptomatic  Female patient, symptomatic | ERT should be considered and is appropriate in all patients at any age of presentation^a^.  Signs/symptoms suggesting major organ involvement, warranting initiation of ERT:  -neuropathic pain, pain crises, Fabry disease neuropathy  -proteinuria/albuminuria NOT attributable to other causes, evidence of renal impairment (may require renal biopsy if isolated)  -stroke or TIA  -symptomatic cardiac disease not due to other causes (dyspnea, palpitations, syncope, chest pain)  -recurrent diarrhea, chronic, disabling GI dysfunction (excluding alternative causes)  -exercise intolerance and impaired sweating |
| Female patient, asymptomatic^b^ | ERT should be considered if there is laboratory, histological, or imaging evidence of injury to the kidney, heart, or the CNS:  -renal disease: decreased GFR (< 90 mL/min/1.73 m^2^ adjusted for age > 40 years [GFR category ≥ G2], persistent albuminuria > 30 mg/g [albuminuria category A2 or A3]), podocyte foot process effacement or glomerulosclerosis on renal biopsy, moderate or severe Gb_3_ inclusions in a range of renal cell types  -silent strokes, cerebral white matter lesions (on brain MRI)  -asymptomatic cardiac disease (cardiomyopathy or arrhythmia, cardiac fibrosis on contrast cardiac MRI)  ERT should also be considered if a skewed X chromosome inactivation pattern with predominant expression of the mutant GLA allele with or without very low AGAL activity have been demonstrated in the presence of signs and symptoms of disease. |
| Later-onset Fabry mutation or missense GLA variance of unknown significance (VUS):  Male and female patients | ERT should be considered and is appropriate if there is laboratory, histological, or imaging evidence of injury to the kidney, heart, or the CNS, as detailed above, even in the absence of typical Fabry symptoms. The abnormalities should be attributable to Fabry disease; this may require histological assessment or biochemical evidence of Gb_3_ accumulation.  The advice of an expert in genetics and management of Fabry disease should be sought for interpretation of the pathogenicity of any VUS.  Individuals with well characterized benign GLA polymorphisms should not be treated with ERT.  In the absence of demonstrable Fabry disease-related tissue pathology or clinical symptoms, ERT may not be appropriate, particularly in heterozygous female patients. These patients should be monitored regularly by a multidisciplinary care team. |
| CNS: central nervous system; ERT: enzyme replacement therapy; AGAL/GLA: α-galactosidase A; GFR: glomerular filtration rate; GI: gastrointestinal; Gb_3_: globotriaosylceramide; MRI: magnetic resonance imaging; TIA: transient ischemic attack; VUS: variant of unknown significance.  ^a^Treatment decisions may be influenced by advanced elderly age of the patient and severe comorbidity.  ^b^Treatment decisions in female patients may be guided by the X chromosome inactivation profile, if assessed. | |

| **Supplemental Table 2: Overview of identified mutations within the recruited patients.** | | |
| --- | --- | --- |
| **mutations** | **missense** | **nonsense/insertions/deletions/intronic** |
| **newly ERT-treated** | p.M42V, p.L45P, p.C63Y, p.G138R, p.A160P, p.C202Y, p.S247P, p.G261R, p.I317T, p.I319T, p.G328R, p.R342Q, p.L344P, p.S345P | p.Y151X, p.Y173X, p.W204X, p.Y216X, p.R220X, p.Q280X, p.T287X, p.R301X  p.T340X, p.R342X, p.W349X, p.Q357X  IVS2 +1G>T, c.369+1G>T, c.714ins7b  c.723dupT, c.744_745del, c.762ins339bp, c.1167dupT, c.1168ins T, Deletion Exon 5-7 |
| **long-term ERT-treated** | p.M42T, p.E66K, p.G138R, p.T194I, p.S247P, p.P265S, p.D266Y, p.N278K, p.A288D, p.R301Q, p.S345P, p.A350P, p.L388P | p.R220X, p.R227X, p.W262X, p.W340X, p.R342X  IVS2+1G>A, c.364delA, c.370-2A>G, p.719delAA, c.744-745 delTA, c.762ins282bp, c.1232delG, c.1167dupT, c.1168insT |
| **untreated (ERT-naïve)** | p.A20P, p.G35E, p.R112C, p.R112H, p.F113L, p.R118C, p.N139S, p.A160P, p.T194I, p.K213M, p.N215S, p.S235Y, p.I242V, p.R252T, p.G274D  p.N278K, p.M296V, p.R301P, p.R301Q, p.E341K, p.R342Q, p.S345P, p.R356T, p.R363C | p.R220X, p.W262X, p.W287X, p.R301X, p.W349X  c.114del, c.702_709del, c.718_719del, c.743_744del, c.744_745del, c.1055_1057dupCTA, c.548-2A>C, c.639+865A>G |
| ERT: enzyme replacement therapy (includes treatment with agalsidase-alfa or -beta) | | |

| **Supplemental Table 3: Quality control of assessed data for analyzed patients (n=159).** | | |
| --- | --- | --- |
| **organ/domain** | **investigation/ measure, n (%)** | **combined work-Up (%)** |
| **medical history** | age: 159 (100.0); BMI: 155 (97.5); SBP: 153 (96.2); DBP: 153 (96.2); ERT status: 159 (100.0) | 98.0 |
| **laboratory parameters** | genotype: 159 (100.0); plasma lyso-Gb3: 125 (68.6) | 84.3 |
| **concomitant medication** | RAAS blockers: 146 (91.8); diuretics: 148 (93.1); analgesics: 145 (91.2); antidepressants: 111(69.8) | 86.5 |
| **clinical presentation** | angiokeratoma: 154 (96.8); edema: 155 (97.5); gastrointestinal symptoms: 122 (76.7); cornea verticillata: 86 (54.1); tinnitus: 120 (75.5); hypakusis: 121 (76.1); FD-related pain: 159 (100.0); fatigue: 115 (72.3); ever stroke/TIA: 158 (99.4); MSSI scores: 148 (93.1); DS3 scores: 142 (89.3) | 84.6 |
| **cardiac measures** | IVSd: 137 (86.2); pacemaker: 156 (98.1); ICD: 156 (98.1), myocardial infarction: 159 (100.0) | 95.6 |
| **renal measures** | ACR: 127 (79.9); serum creatinine: 154 (96.8); eGFR: 154 (96.8); dialysis: 159 (100.0); kidney transplantation: 159 (100.0) | 94.7 |
| **overall data completeness** |  | **90.6** |
| ACR: albumin/creatinine-ratio; DBP: diastolic blood pressure; DS3: Disease Severity Scoring System; eGFR: estimated glomerular filtration rate; FD: Fabry disease; ICD: implantable cardioverter device; IVSd: interventricular septum thickness in diastole; lyso-Gb3: globotriaosylsphingosine; MSSI: Mainz Severity Score Index; NYHA: New York Heart Association; RAAS: renin-angiotensin-aldosterone-system; SBP: systolic blood pressure; TIA: transitory ischemic attack. | | |

| **Supplemental Table 4: Overview of the frequency of organ involvement justifying treatment with enzyme replacement therapy (ERT) according to current guidelines.** | | | |
| --- | --- | --- | --- |
|  | **newly ERT-treated [n=47]** | **long-term ERT-treated [n=41]** | **untreated [n=71]** |
| **single organ involvement** | **13 (27.6)** | **14 (34.1)** | **28 (39.4)** |
| pain | 8 (61.5) | 11 (78.6) | 12 (42.8) |
| renal | 2 (15.4) | 2 (14.3) | 8 (28.6) |
| cardiac | 2 (15.4) | 1 (7.1) | 0 (0.0) |
| CNS | 1 (7.7) | 0 (0.0) | 1 (3.6) |
| GI | 0 (0.0) | 0 (0.0) | 7 (25.0) |
| **multi organ involvement** | **29 (61.7)** | **23 (56.1)** | **24 (33.8)** |
| **2 domains** |  |  |  |
| GI+pain | 5 (17.2) | 2 (8.7) | 9 (37.5) |
| pain+renal | 5 (17.2) | 2 (8.7) | 2 (8.3) |
| cardiac+renal | 4 (13.8) | 1 (4.3) | 4 (16.7) |
| pain+cardiac | 3 (10.3) | 6 (26.1) | 0 (0.0) |
| CNS+renal | 1 (3.4) | 0 (0.0) | 0 (0.0) |
| pain+CNS | 0 (0.0) | 3 (13.0) | 1 (4.2) |
| CNS+cardiac | 0 (0.0) | 2 (8.7) | 1 (4.2) |
| GI+cardiac | 0 (0.0) | 0 (0.0) | 1 (4.2) |
| GI+renal | 0 (0.0) | 0 (0.0) | 1 (4.2) |
| *sum* | *18 (62.1)* | *16 (69.6)* | *19 (79.2)* |
| **3 domains** |  |  |  |
| GI+pain+cardiac | 4 (13.8) | 0 (0.0) | 0 (0.0) |
| pain+cardiac+renal | 3 (10.3) | 2 (8.7) | 1 (4.2) |
| pain+cns+renal | 1 (3.4) | 1 (4.3) | 0 (0.0) |
| GI+pain+CNS | 0 (0.0) | 1 (4.3) | 0 (0.0) |
| GI+CNS+renal | 0 (0.0) | 0 (0.0) | 1 (4.2) |
| GI+pain+renal | 0 (0.0) | 0 (0.0) | 2 (8.3) |
| GI+cardiac+renal | 0 (0.0) | 1 (4.3) | 0 (0.0) |
| *sum* | *8 (27.6)* | *5 (21.7)* | *4 (16.7)* |
| **4 domains** |  |  |  |
| GI+pain+CNS+cardiac | 1 (3.4) | 0 (0.0) | 1 (4.2) |
| pain+CNS+cardiac+renal | 1 (3.4) | 2 (8.7) | 0 (0.0) |
| *sum* | *2 (6.9)* | *2 (8.7)* | *1 (4.2)* |
| **5 domains** |  |  |  |
| GI+pain+CNS+cardiac+renal | 1 (3.4) | 0 (0.0) | 0 (0.0) |
| *sum* | *1 (3.4)* | *0 (0.0)* | *0 (0.0)* |
| Cardiac: left ventricular hypertrophy, Renal: eGFR <90 ml/min/1.73 m^2^, CNS: central nervous system including stroke/ transient ischemic attack, Pain: neuropathic/FD-related pain, GI: gastrointestinal symptoms (diarrhea, abdominal pain). | | | |

| 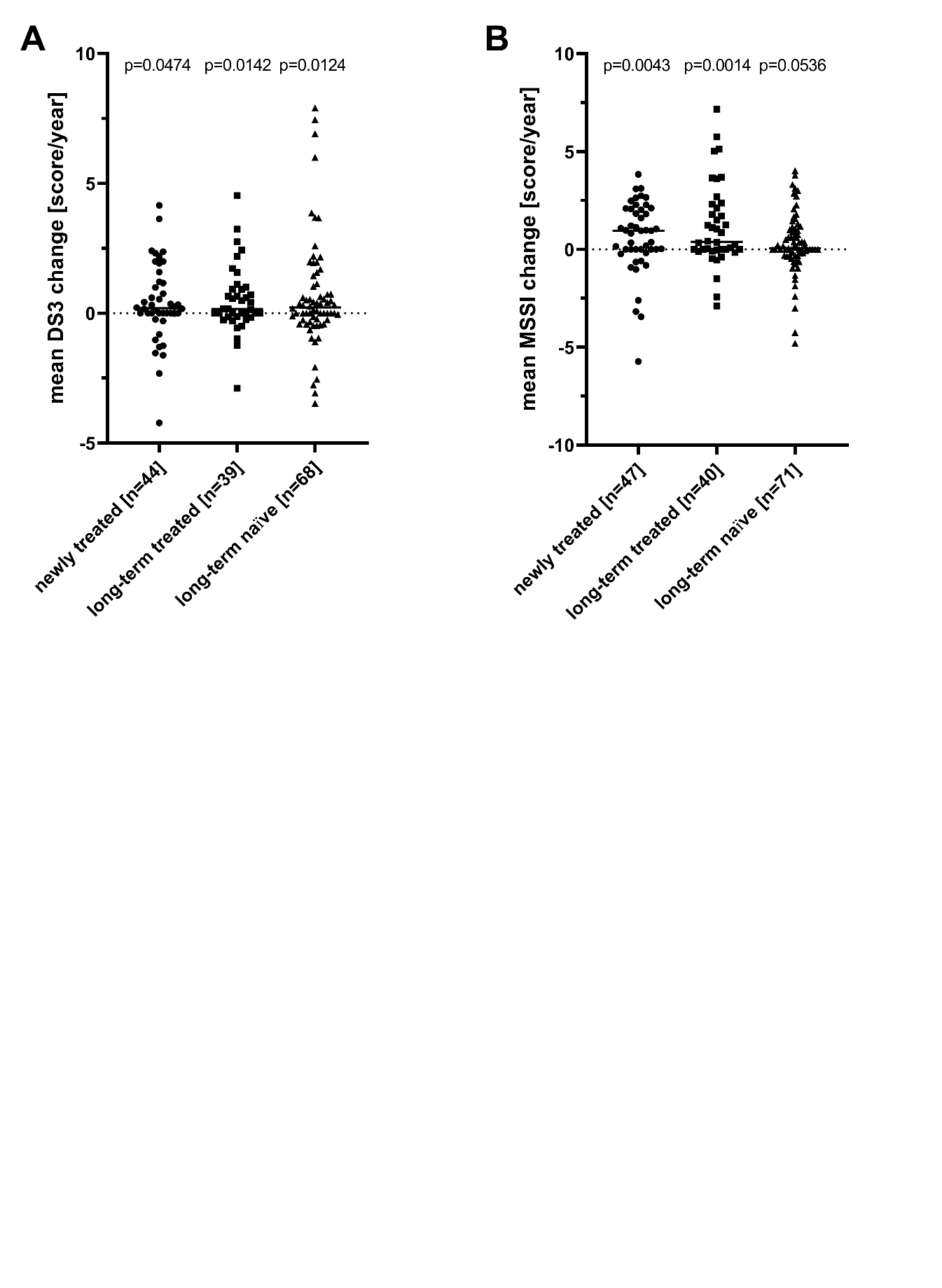 |
| --- |
| **Supplemental Figure 1: Mean disease severity score changes over time. A**) Yearly Disease Severity Scoring System (DS3) changes. **B)** Yearly Mainz Severity Score Index (MSSI) changes. |
